# Mimicking natural polymorphism in *eIF4E* by CRISPR‐Cas9 base editing is associated with resistance to potyviruses

**DOI:** 10.1111/pbi.13096

**Published:** 2019-03-05

**Authors:** Anna Bastet, Delyan Zafirov, Nathalie Giovinazzo, Anouchka Guyon‐Debast, Fabien Nogué, Christophe Robaglia, Jean‐Luc Gallois

**Affiliations:** ^1^ GAFL INRA Montfavet France; ^2^ Laboratoire de Génétique et Biophysique des Plantes CEA CNRS BIAM Aix Marseille University Marseille France; ^3^ Institut Jean‐Pierre Bourgin INRA AgroParisTech CNRS Université Paris‐Saclay Versailles France

**Keywords:** CRISPR‐Cas9, base editing, cytidine deaminase, eIF4E, potyvirus, *Arabidopsis thaliana*

## Abstract

In many crop species, natural variation in eIF4E proteins confers resistance to potyviruses. Gene editing offers new opportunities to transfer genetic resistance to crops that seem to lack natural *eIF4E* alleles. However, because eIF4E are physiologically important proteins, any introduced modification for virus resistance must not bring adverse phenotype effects. In this study, we assessed the role of amino acid substitutions encoded by a *Pisum sativum eIF4E* virus‐resistance allele (W69L, T80D S81D, S84A, G114R and N176K) by introducing them independently into the *Arabidopsis thaliana eIF4E1* gene, a susceptibility factor to the *Clover yellow vein virus* (ClYVV). Results show that most mutations were sufficient to prevent ClYVV accumulation in plants without affecting plant growth. In addition, two of these engineered resistance alleles can be combined with a loss‐of‐function *eIFiso4E* to expand the resistance spectrum to other potyviruses. Finally, we use CRISPR‐nCas9‐cytidine deaminase technology to convert the Arabidopsis *eIF4E1* susceptibility allele into a resistance allele by introducing the N176K mutation with a single‐point mutation through C‐to‐G base editing to generate resistant plants. This study shows how combining knowledge on pathogen susceptibility factors with precise genome‐editing technologies offers a feasible solution for engineering transgene‐free genetic resistance in plants, even across species barriers.

## Introduction

Building resistance to pathogens in plants is a challenge that constantly requires improved technology and new resources. Natural resistance, largely used in conventional breeding, are limited sources, because domestication often reduced the genetic variability of cultivated species and created a bottleneck preventing further improvement (Sikora *et al*., 2011). Natural diversity is however an important reservoir of useful traits that should be developed (Brozynska *et al*., 2016; Zhang *et al*., 2017), and the steady progress in biotechnology can help extend natural resistance to a new level (Palmgren *et al*., 2015). Genetic techniques allowing the introduction of precise modifications in the genome, such as CRISPR‐Cas9, can help overcome species barriers in a simple way, by copying natural variability from one species to another (Jacob *et al*., 2018). Apart from inducing small indels resulting in gene knock‐outs, the CRISPR‐Cas9 system can be used for the targeted action of nucleotide‐modifying enzymes (Eid *et al*., 2018). A fusion of a nuclease‐dead Cas9 or nickase with cytidine deaminase can target point mutagenesis with high precision and has already been successfully used in several species for gene modification (Komor *et al*., 2016; Lu and Zhu, 2017). In plants, manipulation of the CRISPR‐Cas9 system has led to the engineering of herbicide resistance driven by a single mutation in the *Acetolactate synthase* (*ALS*) gene into rice, watermelon and Arabidopsis (Chen *et al*., 2017; Shimatani *et al*., 2017; Tian *et al*., 2018). Similarly, genes that regulate hormone signalling, *DELLA* and *ETR1*, have been modified by introducing point mutations with potential agronomic interest (Shimatani *et al*., 2017). Likewise, base‐editing technologies can be applied to design resistance to pathogens in plants (Borrelli *et al*., 2018; Langner *et al*., 2018; Zaidi *et al*., 2018). Base‐editing technologies are particularly suitable for engineering susceptibility factors, that is, host factors responsible for the infection and proliferation of pathogens. It is known that the modification or suppression of these factors can drive passive and recessive resistance, but due to their role in plant physiology, knocking them out can be associated with adverse developmental phenotypes (Hashimoto *et al*., 2016; Pavan *et al*., 2010; van Schie and Takken, 2014).

The susceptibility factor eIF4E is a perfect candidate for testing biotechnological methods to generate genetic resistance. Natural *eIF4E* resistance alleles have been exploited in breeding for decades and are associated with resistance against a large number of single‐stranded RNA (ssRNA^+^) viruses, mainly belonging to the potyvirus family *Potyviridae* (Robaglia and Caranta, 2006). eIF4E are conserved proteins involved in cap recognition, the first step of eukaryotic mRNA translation. In addition to this important role in translation initiation, eIF4E is also solicited by many viruses for their multiplication, possibly through direct interaction with the viral genome‐linked protein (VPg) of these viruses (Eskelin *et al*., 2011; Hafrén *et al*., 2013; Léonard *et al*., 2000; Michon *et al*., 2006; Wittmann *et al*., 1997). Resistance is caused by amino acid (AA) changes in eIF4E, which are thought to prevent the recognition of eIF4E by the virus without, in most cases, impairing its cellular function (Charron *et al*., 2008). Natural resistance alleles, isolated in economically important crop species, such as lettuce (*Lactuca sativa*), tomato (*Solanum lycopersicum*), pepper (*Capsicum annum*), pea (*Pisum sativum*) and barley (*Hordeum vulgare*), are however absent in other species (Gao *et al*., 2004a; Nicaise *et al*., 2003; Ruffel *et al*., 2002, 2005; Stein *et al*., 2005). Some examples illustrating the potential applications of *de novo eIF4E*‐based resistance are papaya tree (*Carica papaya*), whose production in Hawaii was nearly eradicated by the *Papaya ringspot virus* (PRSV); cassava (*Manihot esculenta*), threatened in Africa by epidemics of the *Cassava brown streak virus* (CBSV); or soybean (*Glycine max*), infected worldwide by the *Soybean mosaic virus* (SMV; Ferreira *et al*., 2002; Hajimorad *et al*., 2018; Patil *et al*., 2015; Rey and Vanderschuren, 2017). Therefore, various groups aimed at knocking out—or down—*eIF4E* and its isoform *eIFiso4E* using a large range of methods, such as insertional mutation, RNAi, EMS mutagenesis and TILLING to generate resistance to ssRNA^+^ viruses in several plants such as *Arabidopsis thaliana* (Duprat *et al*., 2002; Lellis *et al*., 2002), tomato (Mazier *et al*., 2011; Piron *et al*., 2010) and plum (*Prunus domestica*; Wang *et al*., 2013). More recently, *eIFiso4E* and *eIF4E* genes were successfully inactivated using the CRISPR‐Cas9 technique in *A. thaliana* and cucumber (*Cucumis sativa*), respectively (Chandrasekaran *et al*., 2016; Pyott *et al*., 2016), and the inactivation of the atypical eIF4E isoform nCBPs in cassava was associated with reduced susceptibility to CBSV (Gomez *et al*., 2018). Despite their important role in translation initiation, the knock‐out of genes encoding 4E translation initiation factors is often possible due to the high functional redundancy between the different genes of the *eIF4E* family (Patrick and Browning, 2012). Therefore, inactivation of either *eIF4E* or *eIFiso4E* is generally not associated with phenotypic defects Bastet *et al*., 2017). However, because potyviruses are able to selectively use either eIF4E, eIFiso4E or both, knocking out one of these factors leads only to a restricted resistance spectrum (Bastet *et al*., 2018; Duprat *et al*., 2002; Lellis *et al*., 2002; Ruffel *et al*., 2006; Sato *et al*., 2005). A large resistance spectrum can only be conferred by knocking out several 4E genes, thereby profoundly affecting plant development or viability (Bastet *et al*., 2017; Callot and Gallois, 2014; Gauffier *et al*., 2016; Patrick and Browning, 2012). Overall, recent results from different pathosystems indicate that resistance *eIF4E* alleles still encoding functional translation initiation factors should be favoured over loss‐of‐function alleles. The latter are indeed often associated with a limited resistance spectrum and resistance breaking (for review, see Bastet *et al*., 2017). Considering these aspects, it has been suggested that biotechnology‐engineered resistance allele strategies should focus on mimicking natural alleles, whose functionality is not affected, to expand the resistance spectrum without adversely affecting physiological functions (Bastet *et al*., 2017). As a proof‐of‐concept, we recently showed that resistance signatures (AA changes associated with resistance) isolated from the pea (*P. sativum*) *sbm1* allele conferring resistance to *Clover yellow vein virus* (ClYVV; Andrade *et al*., 2009; Gao *et al*., 2004b) can be transferred to the *A. thaliana eIF4E1* gene. This was achieved by complementing an *eIF4E1* knock‐out mutant with a modified *eIF4E1* genomic transgene and assessing the plant development and virus resistance. The resulting allele was associated with resistance to ClYVV and, in combination with another eIF4E‐mediated resistance, provided an expanded resistance spectrum to eight viruses without effects on plant growth or development (Bastet *et al*., 2018).

However, the application of this approach in crops is challenging because the simultaneous introduction of six AA changes in the engineered allele specific to the pea resistance *eIF4E* allele is particularly difficult to achieve via genome editing and extremely unlikely by mutagenesis. Therefore, to understand the relative importance of the six point mutations in resistance, we explored the separate effect of all six mutations independently on resistance and functionality. We show that those polymorphisms are associated with different resistance spectra, mirroring the series of natural *eIF4E* alleles already identified. Interestingly, resistance to ClYVV in *A. thaliana* requires only one or two mutations in *eIF4E*, and these have no adverse effects on plant development. When combined with an *eifiso4e* ^*KO*^ allele, these new alleles expanded the range of resistance spectra to five potyviruses that use eIF4E, eIFiso4E or both. Finally, we introduced the single resistance‐conferring N176K mutation using the CRISPR‐Cas9‐cytidine deaminase editing system in the wild‐type endogenous *eIF4E1* allele. We then showed that this sole mutation was sufficient to produce non‐transgenic resistant plants without affecting growth, thereby mimicking natural variation and providing a proof‐of‐concept of how powerful genome‐editing technology can be used to transfer resistance from a species to another.

## Results

### Assessing the role of independent amino acid substitutions on eIF4E1 protein structure

Natural polymorphisms in *eIF4E* alleles are often associated with resistance to viruses (Robaglia and Caranta, 2006). Several studies have assessed the role of point mutations in either resistance or translation initiation function (Ashby *et al*., 2011; German‐Retana *et al*., 2008; Moury *et al*., 2014). However, it remains difficult to know which AA changes are important for resistance. We previously engineered an *eIF4E1* resistance allele in *A. thaliana* by introducing six non‐synonymous AA substitutions (W69L, T80D, S81D, S84A, G114R, N176K) based on the sequence of pea *sbm1* alleles. These six mutations induced general resistance to potyviruses, but conserved the functionality of eIF4E1 as a translation initiation factor (Bastet *et al*., 2018). Whether all these mutations are necessary to generate a functional resistance allele is unknown, although—in the light of the large *eIF4E* natural allelic series in crops such as pepper—virus resistance is expected to act through different mutational pathways (Moury *et al*., 2014; Poulicard *et al*., 2016). Thus, the determination of the causal mutations may reduce the number of AAs that need to be modified simultaneously, making it easier to engineer *eIF4E* genes in crop plants via gene editing or mutagenesis.

First, we wanted to assess the potential role of each of these mutations for eIF4E function in cell physiology as well as its involvement in virus resistance. To do so, we examined the effect of these mutations independently, with the exception of T80D and S81D which were combined in the same allele due to their vicinity in the sequence. With regard to their AA substitutions, these five alleles were named *eIF4E1* ^*W69L*^, *eIF4E1* ^*T80DS81D*^, *eIF4E1* ^*S84A*^, *eIF4E1* ^*G114R*^ and *eIF4E1* ^*N176K*^.

**Figure 1:**
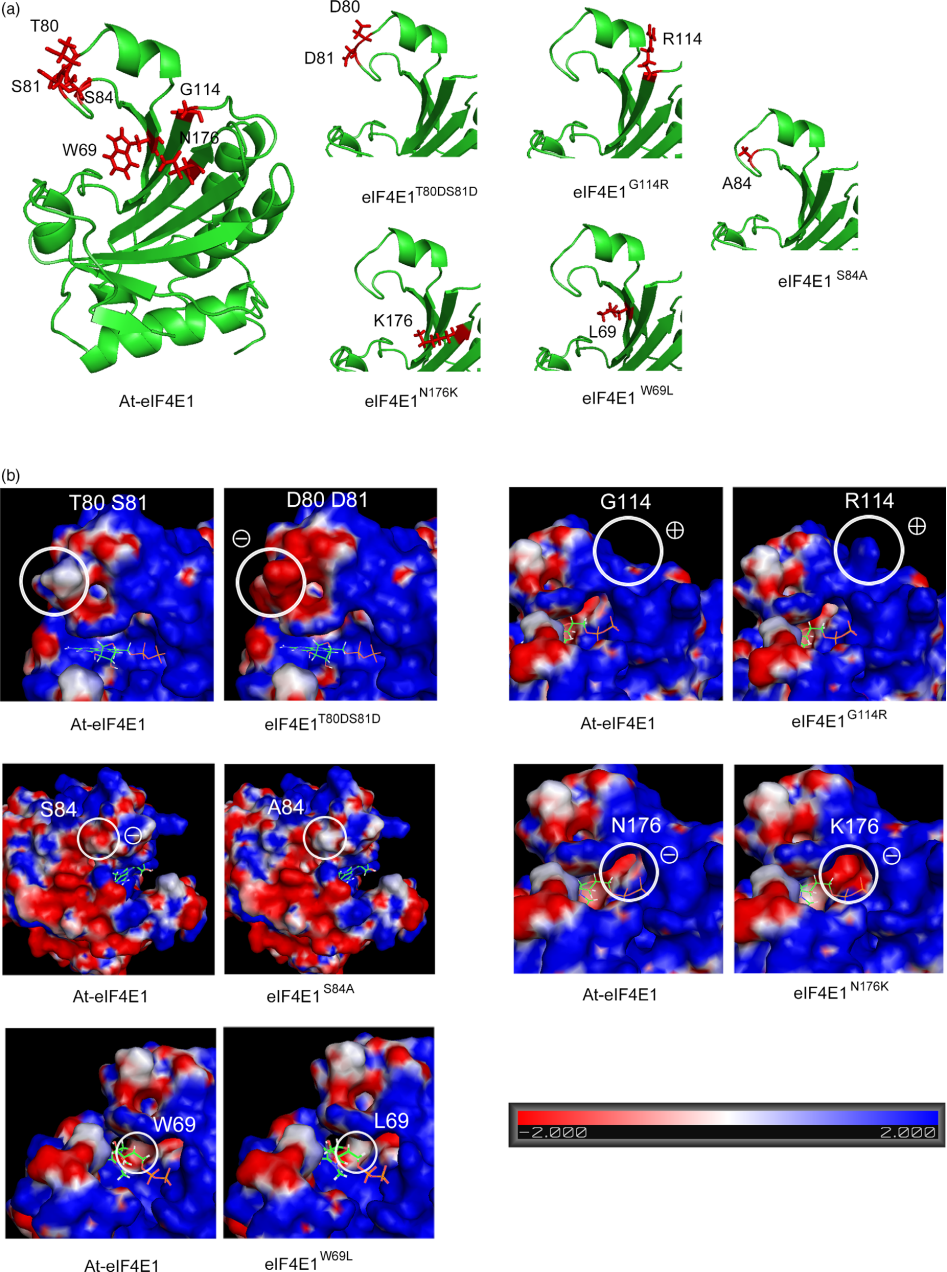
Three‐dimensional analysis of the eIF4E1 proteins encoded by the five constructed alleles *eIF4E1* ^*W69L*^, *eIF4E1* ^*T80*^ ^*DS*^ ^*81D*^, *eIF4E1* ^*S84A*^, *eIF4E1* ^*G114R*^ and *eIF4E1* ^*N176K*^. (a) Three‐dimensional homology modelling of the Arabidopsis eIF4E1 protein, based on crystallography data from the *Pisum sativum* eIF4E 3D structure (PDB ID: 2WMC‐C), for the wild‐type (WT) and the five constructed alleles. The positions of the six amino acids to be introduced are indicated in red along with their side chains. (b) Electrostatic potential of the surface of eIF4E1 proteins compared to the WT. Positions of the amino acid substitutions are circled on the WT protein (left panel) and on the mutated proteins (right panel). To indicate the cap‐binding pocket, a 7‐methyl‐GDP molecule is shown in its binding conformation to the eIF4E protein.

The 3D structures of the proteins encoded by the five alleles were built based on homology modelling using the pea eIF4E as a template (see Methods; Figure 1a). None of the considered mutations induced any changes in the protein backbone, consistent with the surface localization of the modified AAs, suggesting that the overall structure of eIF4E1 protein was conserved in all five alleles. Changes in surface electrostatic potential were also analysed because they can be correlated with the disruption of the interaction between eIF4E and the viral factor VPg (Poulicard *et al*., 2016; Figure 1b). The double T80D‐S81D mutation and the N176K mutation induced clear potential changes from neutral to strongly negative. The effects of the S84A and W69L mutations on electrostatic potential—from negative to neutral charges—were less drastic. Finally, the G114R mutation induced no modification on the electrostatic potential: although glycine is a neutral amino acid, the area surrounding it is highly positive and this positive charge remained after substitution with positively charged arginine. However, G114R was accompanied by a dramatic increase in steric hindrance. Changes in surface hydrophobicity potential were also analysed, and all mutations except W69L induced changes in hydrophobicity on the protein surface (Figure S1).

Overall, the conservation of the structural backbone of the protein suggests that functionality is conserved in the engineered alleles. However, modifications in electrostatic and hydrophobic potentials as well as in steric occupation could affect the protein interaction landscape of eIF4E1.

### *In planta* expression of five alleles encoding functional eIF4E1

**Figure 2:**
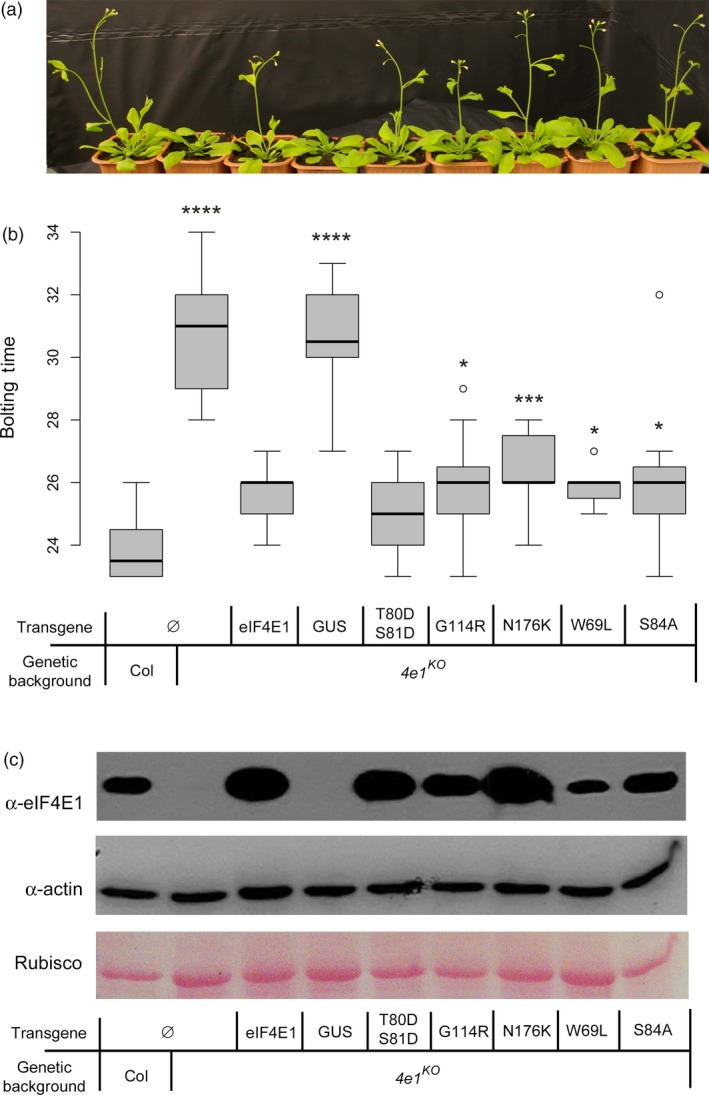
Functional *in planta* complementation of the *eif4e1* knock‐out by the five constructed alleles *eIF4E1* ^*W69L*^, *eIF4E1* ^*T80*^ ^*DS*^ ^*81D*^, *eIF4E1* ^*S84A*^, *eIF4E1* ^*G114R*^ and *eIF4E1* ^*N176K*^. (a) Four‐week‐old plants showing different bolting times. (b) Bolting time in days after sowing assayed on at least 16 plants per genotype. Results for each mutant genotype are pooled from at least two independent lines. Significant differences compared with the wild type, calculated by a Kruskal–Wallis statistical test, are indicated by asterisks for *P* < 0.05 (*), *P* < 0.01 (**), *P* < 0.001 (***) or *P* < 0.0001 (****). (c) Biochemical assay of cap affinity for the five mutated proteins produced. Total protein extracts were Ponceau stained (bottom panel) and exposed to antibodies directed against actin protein (middle panel). Proteins eluted after passing through a cap‐analogue affinity column were exposed to antibodies directed against the Arabidopsis eIF4E1 protein (top panel). Both experiments were repeated at least twice.

These five alleles were constructed *in vitro* using directed mutagenesis on a 3.5 kb fragment comprising the genomic *AteIF4E1*, all introns and a 1.5 kb promoting sequence, before being stably introduced into *A. thaliana eif4e1* ^*KO*^ plants using *A. tumefaciens*‐mediated transformation. An unmodified Arabidopsis *eIF4E1* gene, as well as an unrelated *GUS* gene, were also introduced as positive and negative controls, respectively, in *eif4e1* ^*KO*^ plants (Bastet *et al*., 2018). The correct expression of the transgenes was assessed by reverse‐transcription PCR and western blot analysis on at least two independent lines per construct (Figure S2). As described previously, the complementation of a 7‐day bolting delay associated with *eIF4E1* loss‐of‐function was used as a proxy to assess the functionality of the eIF4E1 proteins encoded by the different alleles (Bastet *et al*., 2018; Figure 2a,b). As observed for the wild‐type control allele, all five alleles, namely *eIF4E1* ^*W69L*^, *eIF4E1* ^*T80DS81D*^, *eIF4E1* ^*S84A*^, *eIF4E1* ^*G114R*^ and *eIF4E1* ^*N176K*^, successfully complemented the bolting delay induced by KO mutation of *eIF4E1*, indicating that they encode functional eIF4E1 proteins. The binding ability of the modified eIF4E1 was also confirmed, with protein extracts from all five types of transgenic plants proving their ability to bind a cap analogue (Figure 2c).

In conclusion, all five engineered alleles encode functional eIF4E1 proteins. This plasticity is consistent with previous assessment of an allelic series of eIF4E leading to virus resistance in pepper, lettuce and pea (Ashby *et al*., 2011; Charron *et al*., 2008; German‐Retana *et al*., 2008).

### Both *eIF4E1* ^*T80DS81D*^ and *eIF4E1* ^*N176K*^ confer full resistance to two isolates of ClYVV

**Figure 3:**
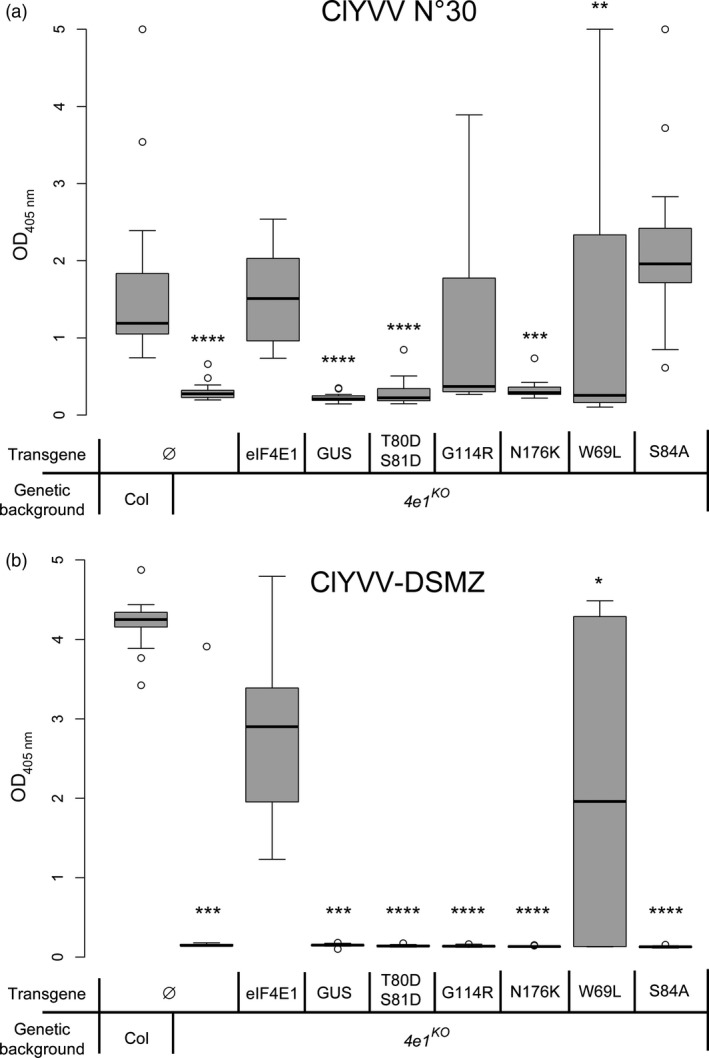
Viral accumulation of ClYVV in *eif4e1* ^*KO*^ plants complemented with constructed alleles assessed using DAS‐ELISA. Viral accumulation of the ClYVV No. 30 (a) and the ClYVV‐DSMZ (b) isolates was assessed using DAS‐ELISA directed against ClYVV. Sixteen plants were tested per genotype. Results for each mutant genotype were pooled from at least two independent lines. Kruskal–Wallis statistical analyses on this data are indicated with asterisks according to the significance of differences from the wild type at *P* < 0.05 (*), *P* < 0.01 (**), *P* < 0.001 (***) or *P* < 0.0001 (****).

The five *eIF4E1* alleles encoding functional variants were then assessed for resistance to ClYVV, a potyvirus relying on eIF4E1 to infect Arabidopsis and that cannot use the protein encoded by *eIF4E1* ^*R*^—the synthetic allele incorporating six AA changes (Bastet *et al*., 2018). All complemented plants and controls were inoculated with ClYVV and viral accumulation was measured 31 days post‐infection using a double antibody sandwich enzyme‐linked assay (DAS‐ELISA) with antibodies directed against ClYVV (Figure 3). Two isolates of ClYVV were used to assay robust resistance alleles: the ClYVV No. 30 isolate used previously (Bastet *et al*., 2018; Sato *et al*., 2005; Takahashi *et al*., 1997) and an isolate provided by the DSMZ company. The *eif4e1* ^*KO*^ plants were significantly resistant to both isolates, whereas wild‐type plants and *eif4e1* ^*KO*^ plants complemented with a wild‐type eIF4E1 were all fully susceptible. *eif4e1* ^*KO*^ *eIF4E1* ^*W69L*^ plants were also susceptible to both isolates, suggesting that the W69L substitution is not involved in resistance to ClYVV. Interestingly, the *eif4e1* ^*KO*^ *eIF4E1* ^*W69L*^ plants' responses to both isolates were not homogenous, with seven plants showing full resistance and nine plants showing full susceptibility. This difference may be caused by resistance breaking, as previously shown for other eIF4E‐based alleles (Charron *et al*., 2008; Lebaron *et al*., 2016). On the contrary, both *eif4e1* ^*KO*^ *eIF4E1* ^*T80DS81D*^ and *eif4e1* ^*KO*^ *eIF4E1* ^*N176K*^ plants displayed full resistance to the No. 30 and DSMZ isolates. Finally, *eif4e1* ^*KO*^ *eIF4E1* ^*S84A*^ and *eif4e1* ^*KO*^ *eIF4E1* ^*G114R*^ plants accumulated the ClYVV No. 30 isolate, but not the ClYVV DSMZ isolate.

These results show that the W69L substitution on its own is not involved in resistance to two isolates of ClYVV, but either the T80D‐S81D combination or the N176K substitution is sufficient to prevent both isolates of ClYVV from recruiting the eIF4E1 protein. On the other hand, both S84A and G114R were associated with a resistance limited to the ClYVV DSMZ isolate. In conclusion, the *eIF4E1* ^*T80DS81D*^ and *eIF4E1* ^*N176K*^ alleles both appeared as functionally resistant alleles to both ClYVV isolates.

### *eIF4E1* ^*T80DS81D*^, *eIF4E1* ^*G114R*^ and *eIF4E1* ^*N176K*^ alleles allow resistance pyramiding with an eIFiso4E null allele at no yield loss, but with different resistance spectra

**Figure 4:**
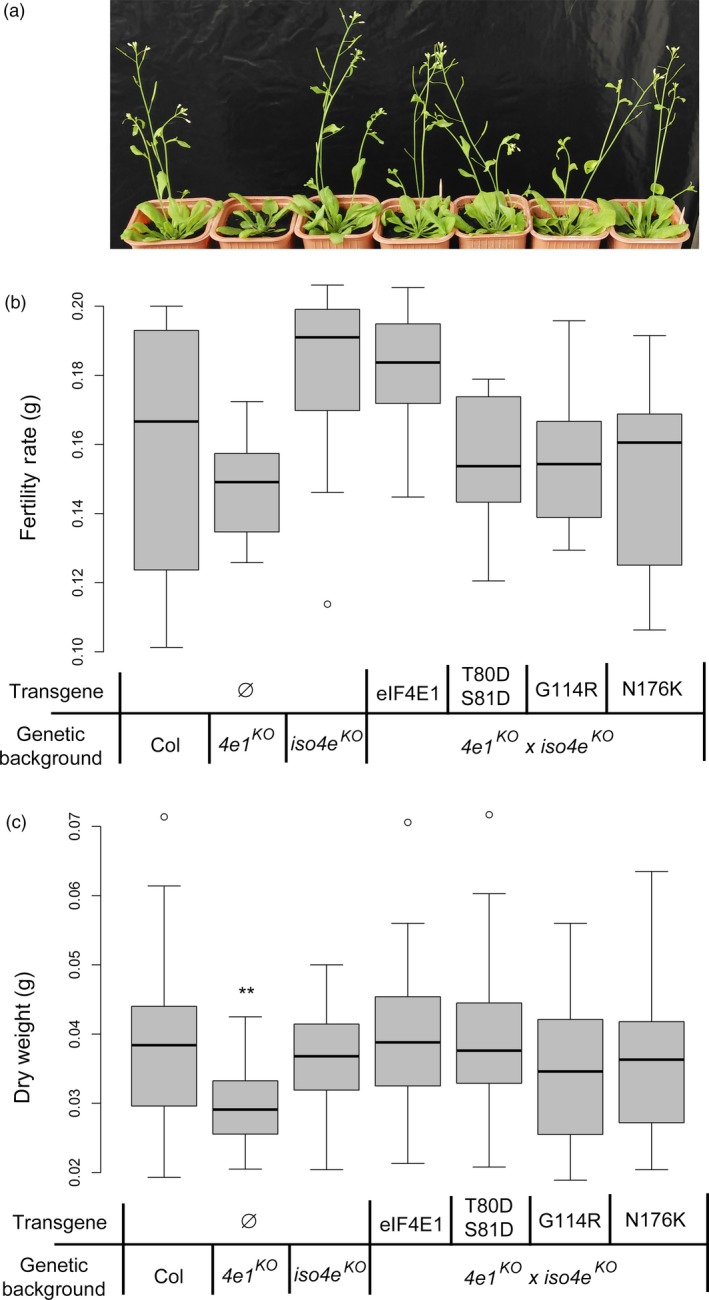
Viability and phenotype assessment of double‐mutant *eif4e1* ^*KO*^ *eifiso4e* ^*KO*^ plants complemented with *eIF4E1* ^*T80*^ ^*DS*^ ^*81D*^, *eIF4E1* ^*G114R*^ or *eIF4E1* ^*N176K*^ alleles. (a) Bolting time delay observed on 4‐week‐old plants. (b) Analysis of fertility rate by weighing total seed production of 10 plants per genotype. (c) Analysis of plant dry weight, results are pooled from 20 plants from at least two independent lines per genotype. Kruskal–Wallis statistical analyses on these data are indicated by asterisks according to the significance of differences from the wild type at *P* < 0.05 (*), *P* < 0.01 (**), *P* < 0.001 (***) or *P* < 0.0001 (****).

We previously showed that, although simultaneous null mutations in both *eIF4E1* and *eIFiso4E* are lethal in Arabidopsis, resistance associated with these two genes can be combined at no yield loss through genetic complementation with the *eIF4E1* ^*R*^ allele designed from pea (Bastet *et al*., 2018; Callot and Gallois, 2014). Moreover, this genetic combination expands the resistance spectrum to new viruses and resistance‐breaking (RB) isolates able to recruit both eIF4E1 and eIFiso4E (Bastet *et al*., 2018). Here, in this study, either *eIF4E1* ^*T80DS81D*^, *eIF4E1* ^*G114R*^ or *eIF4E1* ^*N176K*^ alleles can be introgressed in an *eif4e1* ^*KO*^ *eifiso4e* ^*KO*^ double‐mutant background (see Methods) and rescue its lethality, confirming that these three alleles are functional (Figure 4a). These new lines were named *eif4e1* ^*KO*^ *eifiso4e* ^*KO*^ *eIF4E1* ^*T80DS81D*^, *eif4e1* ^*KO*^ *eifiso4e* ^*KO*^ *eIF4E1* ^*G114R*^ and *eif4e1* ^*KO*^ *eifiso4e* ^*KO*^ *eIF4E1* ^*N176K*^ respectively. Further analysis of the development of these plants showed that these three alleles can rescue the developmental phenotypes associated with a loss‐of‐function in *eIF4E1*, such as delayed bolting time (Figures 4a and S3a), fertility rate (Figure 4b) and plant biomass as assessed by dry and fresh rosette weight (Figures 4c and S3b respectively)**,** confirming that they are fully functional and do not affect development or yield, which are important agronomic traits.

**Figure 5:**
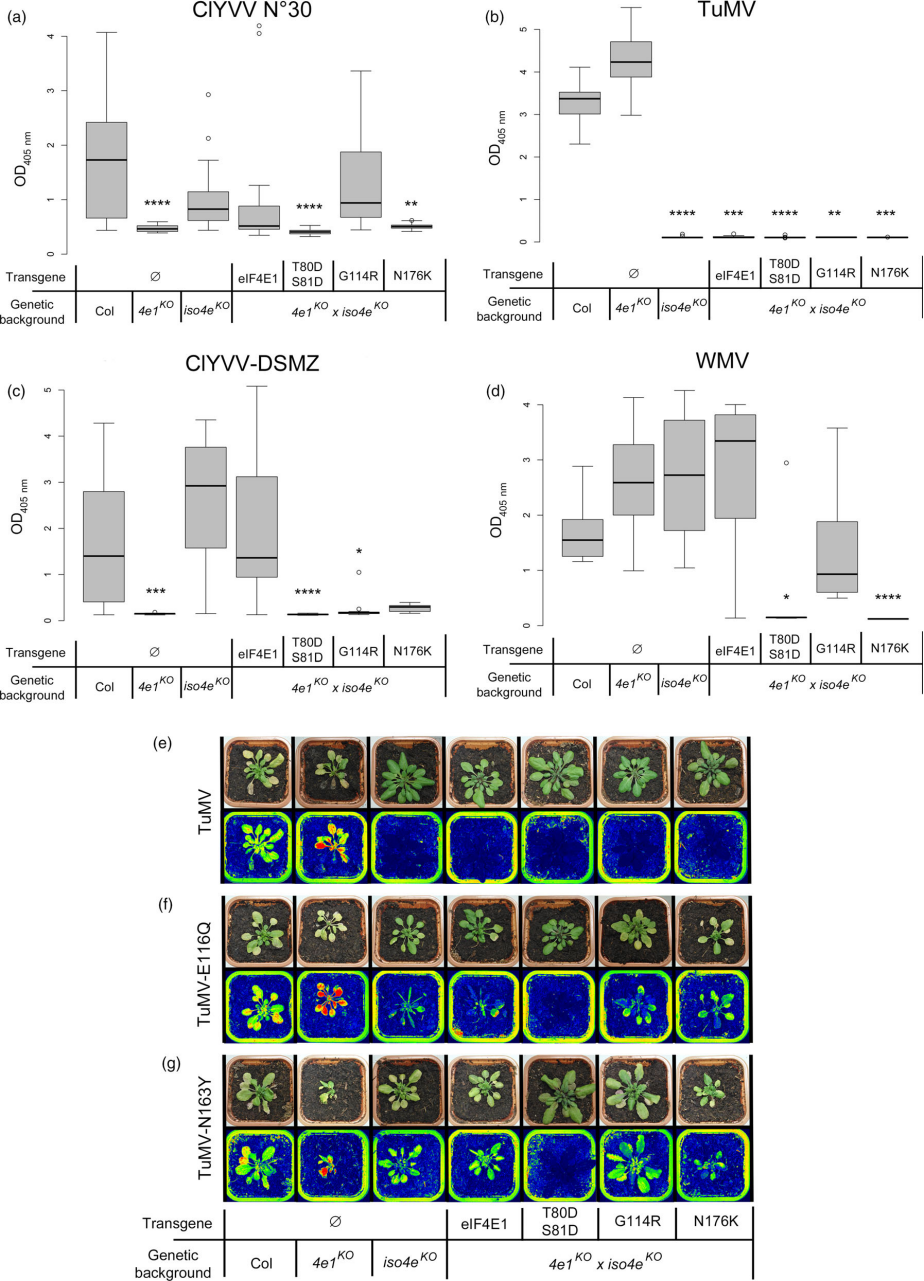
Virus resistance analyses of *eIF4E1* ^*T80*^ ^*DS*^ ^*81D*^, *eIF4E1* ^*G114R*^ or *eIF4E1* ^*N176K*^ alleles in a double‐mutant *eif4e1* ^*KO*^ *eifiso4e* ^*KO*^ background. (a–d) Mutant plants were tested for resistance to ClYVV No. 30 (a), ClYVV‐DSMZ (b), TuMV (c) and WMV (d). Results for each mutant genotype were pooled from at least two independent lines. Sixteen plants were tested per genotype. Kruskal–Wallis statistical analyses on these data are indicated by asterisks according to the significance of the differences from the wild type at *P* < 0.05 (*), *P* < 0.01 (**), *P* < 0.001 (***) or *P* < 0.0001 (****). (e–g) GFP‐Camera fluorescence detection of viral accumulation of GFP‐fused strains of TuMV (e), RB‐TuMV‐E116Q (f) and RB‐TuMV‐N163Y (g). Plants, representative of the twelve plants tested per genotype and per virus isolate, are shown in natural light (top panel) and false GFP colours (bottom panel) for each assay.

Next, the resistance spectrum to potyviruses was assessed to see whether the separate AA substitutions in eIF4E1 were sufficient to span the broad resistance spectrum associated with the pea‐like *eIF4E1* ^*R*^ synthetic allele harbouring six AA changes (Bastet *et al*., 2018). Resistance of *eif4e1* ^*KO*^ *eifiso4e* ^*KO*^ *eIF4E1* ^*T80DS81D*^, *eif4e1* ^*KO*^ *eifiso4e* ^*KO*^ *eIF4E1* ^*N176K*^ and *eif4e1* ^*KO*^ *eifiso4e* ^*KO*^ *eIF4E1* ^*G114R*^ plants to ClYVV No. 30 and DSMZ isolates, *Turnip mosaic virus* (TuMV) and *Watermelon mosaic virus* (WMV) was assessed using DAS‐ELISA following manual inoculations (Figure 5). Resistance to the ClYVV No. 30 or DSMZ isolates was in accordance with above results in the *eif4e1* ^*KO*^ single‐mutant background (see Figure 3): *eIF4E1* ^*T80DS81D*^ and *eIF4E1* ^*N176K*^ were associated with resistance to both isolates, but *eIF4E1* ^*G114R*^ was only associated with resistance to the ClYVV DSMZ isolate (Figure 5a,b). Resistance pyramiding with *eifiso4e* was efficient for all three combinations as attested by the full resistance of all three lines to TuMV, a virus using eIFiso4E (Figure 5c). The resistance to WMV, a virus that can use either eIF4E1 or eIFiso4E mirrors the resistance to ClYVV No. 30: both the *eif4e1* ^*KO*^ *eifiso4e* ^*KO*^ *eIF4E1* ^*T80DS81D*^ and the *eif4e1* ^*KO*^ *eifiso4e* ^*KO*^ *eIF4E1* ^*N176K*^ lines were fully resistant to WMV whereas the *eif4e1* ^*KO*^ *eifiso4e* ^*KO*^ *eIF4E1* ^*G114R*^ line was susceptible (Figure 5d). Finally, we tested the resistance to two TuMV resistance‐breaking (RB) isolates. These isolates can break *eifiso4e* ^*KO*^ resistance because they have gained the ability to recruit eIF4E1 in addition to eIFiso4E (Bastet *et al*., 2018). Plants were agro‐inoculated with either RB‐TuMV‐E116Q or RB‐TuMV‐N163Y viral isolates which express the GFP reporter gene, and viral accumulation was assayed using a GFP camera (Figure 5e–g). Only *eif4e1* ^*KO*^ *eifiso4e* ^*KO*^ *eIF4E1* ^*T80DS81D*^ plants did not accumulate GFP for either RB‐TuMV isolate. The *eIF4E1* ^*T80DS81D*^ allele is therefore an efficient resistance allele to RB‐TuMV isolates, but *eIF4E1* ^*N176K*^ and *eIF4E1* ^*G114R*^ are not.

In conclusion, our results show that single or double mutations in *eIF4E1* provide efficient resistance alleles without impairing plant development. This resistance profile is particularly advantageous in the perspective of using genome‐editing technologies to induce resistance in plants.

### Engineering the N176K substitution using CRISPR‐Cas9‐cytidine deaminase editing produces transgene‐free resistant plants

Base‐editing technologies are efficient tools for designing alleles without transgenesis (Eid *et al*., 2018; Hess *et al*., 2017). Recently, the CRISPR‐Cas9‐cytidine deaminase system has allowed precise editing in alleles of Arabidopsis, rice and tomato (Chen *et al*., 2017; Li *et al*., 2017; Lu and Zhu, 2017; Shimatani *et al*., 2017). In this system, Cas9 nuclease inactivated at one of its catalytic sites (D10A mutation; i.e. a nickase), is fused to a cytidine deaminase enzyme to direct the conversion of cytosine to thymine (Shimatani *et al*., 2017). In addition, an error‐prone mechanism can also convert the modified base to guanine (G) or adenine (A) (Nishida *et al*., 2016).

**Figure 6:**
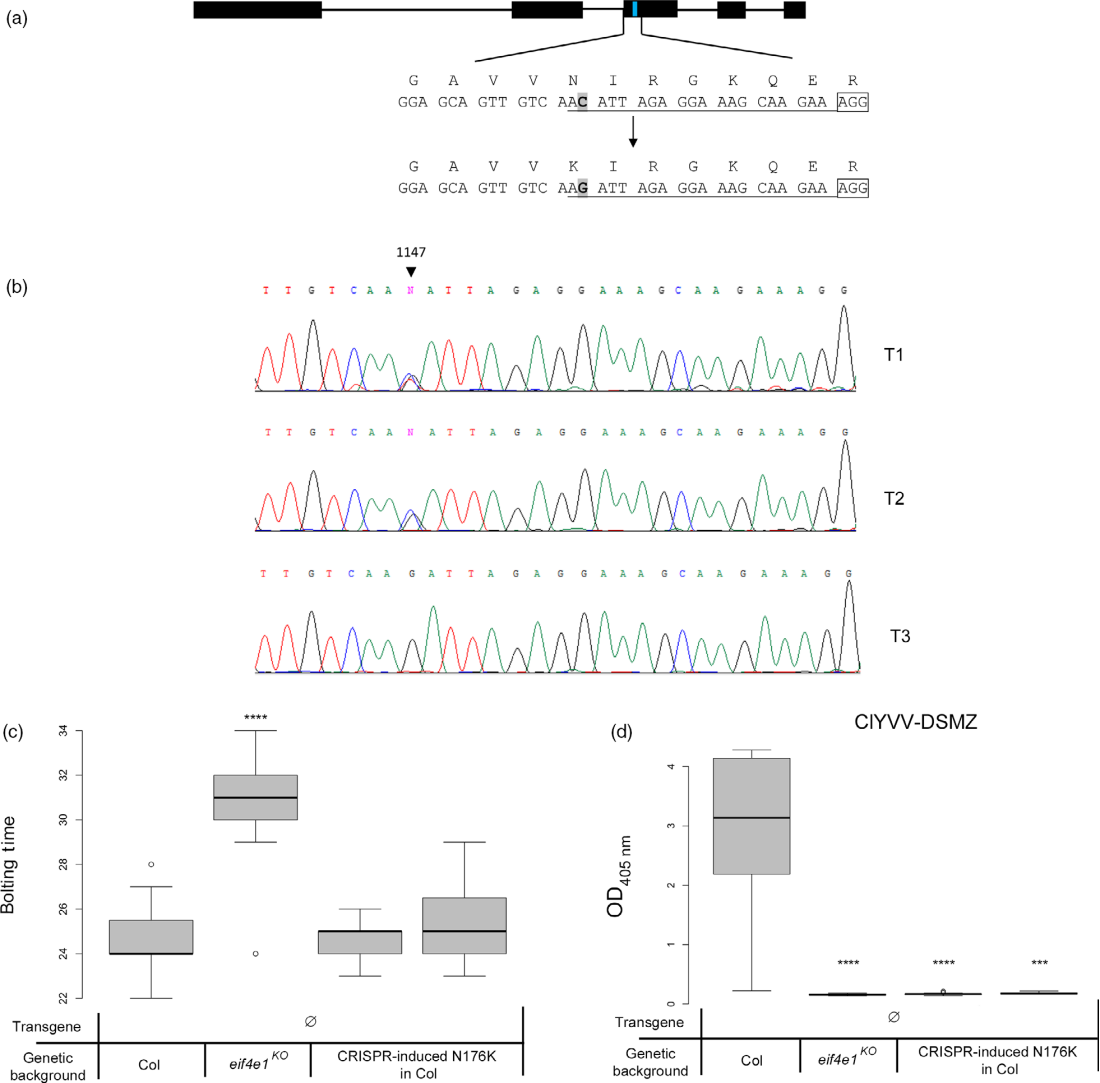
Use of CRISPR‐nCas9‐cytidine deaminase fusion on wild‐type plants to obtain transgene‐free plants containing the N176K mutation. (a) Diagram of the Arabidopsis *eIF4E1* gene. Black boxes indicate exons and black lines introns, both are to scale. The blue line indicates the position of the mutation to introduce. The target sequence is shown underneath the diagram along with the expected base change; the corresponding AAs are shown above the sequence. The PAM is boxed and the sgRNA sequence is underlined. (b) Chromatogram from sequencing data on T1, T2 and T3 plants showing polymorphism at the target site. (c) Results of the bolting time assay in days after sowing on T4 transgene‐free plants homozygous for the N176K mutation. (d) ClYVV‐DSMZ accumulation assessed using DAS‐ELISA on modified transgene‐free T4 N176K plants. Sixteen plants per genotype were tested. Kruskal–Wallis statistical analyses on these data are indicated by asterisks according to the significance of differences from the wild type at *P* < 0.05 (*), *P* < 0.01 (**), *P* < 0.001 (***) or *P* < 0.0001 (****).

Sequence assessment revealed that the simultaneous T80D and S81D amino acid changes, requiring four nucleotide changes, could not be achieved using current base‐editing tools, although *eIF4E1* ^*T80DS81D*^ is the most efficient resistance allele. In contrast, the N176K mutation could be potentially obtained by C‐to‐A or C‐to‐G conversions from the N176‐encoding triplet located 5′ upstream from a potential protospacer adjacent motif (PAM) AGG (Figure 6a). Upon deamination, a C‐to‐T transition result in a synonymous substitution (N176N) and the less frequent C‐to‐G or C‐to‐A transversions result in the expected N176K modification. We thus aimed at introducing the C‐to‐G or C‐to‐A transversion leading to the N176K mutation to convert the wild‐type genomic allele into a virus‐resistance allele.

A 20‐nt single guide RNA (sgRNA) was designed covering the position of the N176 codon at the beginning of *eIF4E1* exon 3 (position +1146 to +1165 of the genomic sequence relative to the ATG): the targeted cytidine, at +1147, is located at position −19 upstream of the PAM sequence (NGG) required for binding the nuclease (Figure 6a). This sgRNA was cloned into a plasmid containing the nCas9^At^‐PmCDA1^At^ construction provided by (Shimatani *et al*., 2017) and transformed into wild‐type Col‐0 plants. To assess the efficiency of editing of the targeted region, genomic DNA was extracted from six independent T1 leaf samples and the targeted *eIF4E1* region was amplified and bulk sequenced: three plants of the six showed high polymorphism at the targeted C site only, showing the activity of the deaminase in independent cells (Figure 6b). The amplitude of the chromatogram peaks corresponding to modified nucleotides compared with the wild‐type cytosine peak in the analysed T1 plants confirmed the efficiency of editing via the Cas9‐cytidine deaminase construction previously observed (Shimatani *et al*., 2017). For further analysis, the amplified fragments were subcloned and independently sequenced. Of the 28 sequencing reads, 19 had the target C substituted with G or A. Among those 19 alleles, six of them displayed additional mutations or indels in addition to the C substitution. Among the 13 alleles showing only modification of the targeted C, the C‐to‐G substitution seemed highly favoured compared to the C‐to‐A substitution, observed in only one sequence. Surprisingly, the most expected modification, the C‐to‐T substitution, was not observed among this limited set of plants, although it was observed in subsequent experiments (Table S1).

The three T1 plants with the expected base changes were allowed to self and T2 plants were sown before being collected and sequenced to check if the mutations were stably transmitted to the next generation. At the same time, the presence of the transgene cassette was assessed by PCR screening to identify transgene‐free plants (see Methods). The *eIF4E1* target site was sequenced and three T2 plants heterozygous for the C1147G nucleotide substitution (Figure 6b) were selected and selfed. Two T3 transgene‐free lines homozygous for the C1147G mutation were selected and selfed for further experiments on T4 plants. The correct accumulation of both mRNA and protein expressed from the edited *eIF4E1* gene was confirmed in these T4 plants (Figure S4).

A flowering time assay on these T4 plants was performed to ensure the physiological functionality of the protein produced by the mutated allele. All modified lines displayed a bolting time similar to the wild type (Figure 6c). Finally, we showed that the T4 CRISPR‐edited plants were fully resistant to the ClYVV DSMZ isolate (Figure 6d), confirming that the precise edition of the *eIF4E1* gene turns it into a functional resistance gene in a transgene‐free manner.

## Discussion

In this work, we dissected the *eIF4E1* polymorphisms responsible for resistance to potyviruses. Five alleles harbouring independent mutations (*eIF4E1* ^*W69L*^, *eIF4E1* ^*T80DS81D*^, *eIF4E1* ^*S84A*^, *eIF4E1* ^*G114R*^ and *eIF4E1* ^*N176K*^) were assessed for both functionality and capacity to confer virus resistance, alone or in combination with a loss‐of‐function of the isoform eIFiso4E. We showed that the non‐synonymous polymorphisms N176K and T80D S81D in *eIF4E1* are sufficient to induce resistance to potyviruses without affecting plant physiology. Finally, using the CRISPR/Cas9n‐cytidine deaminase system, we were able to directly introduce the N176K mutation in wild‐type Arabidopsis and generate transgene‐free resistant plants carrying an allele that mimics polymorphism naturally found in other species.

**Figure 7:**
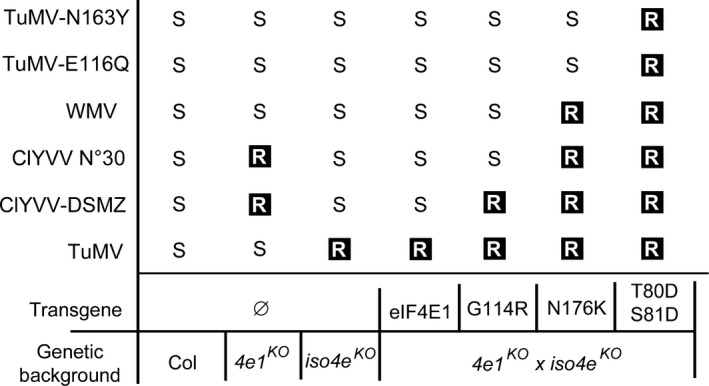
Resistance spectra of the *eif4e1* ^*KO*^ *eifiso4e* ^*KO*^ lines complemented with *eIF4E1* ^*T80*^ ^*DS*^ ^*81D*^, *eIF4E1* ^*G114R*^ or *eIF4E1* ^*N176K*^ alleles when challenged with various potyviruses. R, resistant; S, susceptible.

By separately analysing each mutation from a natural resistance allele, we wanted to assess the role of each mutation in resistance and determine the minimal number of AA changes needed to achieve resistance in plants. Usually, the effect of AA changes can be assessed by the analysis of natural allelic series or by overexpression studies in a resistant background, as has been done for several studied pathosystems (Ashby *et al*., 2011; Charron *et al*., 2008; German‐Retana *et al*., 2008; Yang *et al*., 2017). Our conclusions were consistent with and confirm previous studies (Ashby *et al*., 2011; German‐Retana *et al*., 2008; Kim *et al*., 2014), showing that most mutations selected among those found naturally do not affect the role of eIF4E factor in translation initiation. In contrast, the mutated alleles were associated with different spectra of resistance to the viruses tested. These patterns seem to follow the ‘game of mirrors' of resistance/susceptibility between natural *eIF4E* alleles and potyvirus isolates exemplified by the *Capsicum* spp. PVY and TEV pathosystems (Charron *et al*., 2008; Moury *et al*., 2014; Figure 7). It also shows that, as expected from analysis of natural alleles, there are different pathways to resistance.

Mutations in eIF4E involved in resistance to potyviruses are generally localized in two regions of the protein: the first region (Region I) is positioned near—and partially overlaps—the cap‐binding pocket and the second (Region II) is next to the cap‐binding site, facing Region I (Monzingo *et al*., 2007; Robaglia and Caranta, 2006). Here, we found that the T80D‐S81D mutations, located in Region I, are the most efficient, associated with broad resistance to all isolates assayed. The AAs corresponding to these mutations are highly exposed on the eIF4E protein surface and are associated with changes in electrostatic and hydrophobic potential, as shown for mutations associated with natural resistance (Poulicard *et al*., 2016). Consistently, the corresponding A73D‐A74D changes were also found in the pepper *pvr2* ^*6*^ natural resistance allele. The independent selection of these mutations in pepper and in pea provides a compelling argument for their contribution to potyvirus resistance (Charron *et al*., 2008). Interestingly, the G114R mutation located in Region II was not sufficient to provide efficient resistance, similar to equivalent mutations in the same region in the *Solanum pimpinellifolium* LA0411 resistance allele (Lebaron *et al*., 2016). T80D‐S81D and G114R are substitutions that are frequently isolated in resistance alleles in pepper, tomato and pea, and appear to be under positive selection (Cavatorta *et al*., 2008; Moury *et al*., 2014). In addition to these observations, we identified N176K as an important mutation lying outside of Regions I and II. The location of this mutation was unexpected, because to date no resistance to potyviruses has been characterized outside Regions I and II in natural alleles. Our studies enhance the concept of a conserved signature associated with eIF4E‐mediated resistance to potyviruses. These mutations can be used as a blueprint to engineer *de novo eIF4E*‐based resistance in crop plants.

Our results raise the question of the reason why several mutations have been selected for in the pea allele, whereas only one or two seem to suffice. Although such variability may result from genetic linkage rather than true selection, the accumulation of several mutations in this particular area, as observed for different species, suggests that it is most likely not coincidental. In fact, natural resistance alleles are often characterized by numerous mutations. In pepper, in which a wide series of virus‐resistance allelic variations in *eIF4E1* have been identified, several studies have evaluated the selection processes that lead to such variability (Charron *et al*., 2008; Moury *et al*., 2014; Poulicard *et al*., 2016). Sequence analyses of 25 natural *pvr2* (i.e. *eIF4E1*) resistance alleles helped recreate their mutational pathways explaining the co‐evolution with potyviruses. Additional mutations on a resistance allele can lead to resistance pyramiding against several viruses, thus expanding the resistance spectrum and/or increasing resistance durability (Charron *et al*., 2008; Moury *et al*., 2014; Poulicard *et al*., 2016; Yeam *et al*., 2007).

In this work, CRISPR‐Cas9 cytidine deaminase was successfully used to introduce the N176K mutation in the *eIF4E* gene and confer a transgene‐free resistance to ClYVV. The use of this method in plant breeding is in its very first stages and not many studies have explored the potential of its effectiveness in plant breeding projects. Here, we showed that this genome‐editing system can be used with high efficiency without much equipment: we screened only six plants to isolate the desired mutation. We obtained 50% of modified T1 plants, which are quite high compared with previous studies (Chen *et al*., 2017). Further improvements are sure to increase the adaptability of the system to several applications. For example, adenine base editors are currently being developed in a CRISPR/Cas9 fusion system, enabling A‐to‐G modifications (Gaudelli *et al*., 2017; Li *et al*., 2018) and improvements in PAM variability to increase target possibilities are the focus of other studies (Anders *et al*., 2016; Hu *et al*., 2018; Kaya *et al*., 2016; Murovec *et al*., 2017; Nishimasu *et al*., 2018; Steinert *et al*., 2015). Multiplex CRISPR‐Cas9 modifications, targeting several sites at the same time is also a promising aspect of this technique (Shimatani *et al*., 2018; Wang *et al*., 2018; Xu *et al*., 2016). These advances provide important tools that may lead to the application of the proof‐of‐concept developed here to crop species (Langner *et al*., 2018).

In conclusion, our study proves that natural variation bringing resistance in one species can efficiently work in another. We showed that one nucleotide modification is sufficient to confer resistance and that this modification can be successfully introduced using the CRISPR/Cas9 base‐editing system, thus highly simplifying the transfer process (Hess *et al*., 2017). More generally, this technique can potentially be applied to all types of genetic resistance relying on susceptibility factors in plants, regardless of the type of pathogen. Nematodes, fungi and bacteria, as well as viruses, need to hijack host factors to infect plants, and these are all potential sources of resistance to be explored (Pavan *et al*., 2010). This study demonstrates that using base‐editing technology can efficiently transfer signature resistance mutations from one species to another. Such fine‐tuned editing opens new opportunities for breeding resistance in plants.

## Methods

### Plant materials and growth conditions

The wild‐type genotype used throughout this study was the Columbia‐0 Arabidopsis accession (Col‐0). *eif4e1* ^*KO*^ and *eifiso4e* ^*KO*^ lines carry the homozygous knock‐out for, respectively, the *eIF4E1* gene At1g18040 (T‐DNA insertion line SALK_145583) and the *eIFiso4E* gene At5g3560 (transposon dSpm insertion line; Duprat *et al*., 2002) in the Col‐0 background. Genetic crosses between genotypes were carried out manually by cross‐pollination of emasculated immature flowers. Genotyping was done using the primers listed in Table S2.

For *in vitro* growth, seeds were sterilized with a 95% ethanol‐0.1% Tween solution and sown onto plates containing Murashige and Skoog (MS) medium (Sigma‐Aldrich, St Louis, MO), supplemented with 5 mg/L hygromycin B when selection was needed. After 2 weeks on plates, plantlets were transferred to pots filled with soil in a culture chamber at 20 °C (night) and 24 °C (day) temperature, with a 16 : 8 h light:dark periodicity for bolting time assays and 8 : 16 h light:dark periodicity for virus resistance assays. For dry weight, fresh weight and fertility rate assays, seeds were sown directly in soil and pots were randomized on the culture chamber shelf.

### Three‐dimensional protein structure modelling, electrostatic and hydrophobic potentials

Homology modelling of the wild‐type and mutated Arabidopsis eIF4E proteins were carried out using the YASARA software (http://www.yasara.org/), using structural data from pea (*P. sativum*) eIF4E (GenBank ID: AY423375, PDB ID: 2WMC‐C) as the template. Protein structure and surface were visualized using PyMol software (https://pymol.org/). Electrostatic potential was calculated using the APBS and PDB2PQR plugins in Pymol (http://www.poissonboltzmann.org/). Hydrophobicity was shown on protein surface using Pymol using a colour code based on the Eisenberg's hydrophobicity scale (Eisenberg *et al*., 1984).

### Plasmid construction and plant transformation

Construction and cloning of *eIF4E* mutated alleles were done as described previously on a 3533 bp genomic–At4g18040—*eIF4E1* fragment (spanning 1500 bp of the promoter region and 150 bp of the 3′UTR; Bastet *et al*., 2018). Site‐directed mutagenesis associated with six AA changes (W69L, T80D, S81D, S84A, G114R and N176K) were independently introduced with the QuikChange II Site‐Directed Mutagenesis Kit (Stratagene, La Jolla, CA) using the primers listed in Table S2 and subcloned into pDONR207 using Gateway™ BP recombination (Invitrogen, Carlsbad, CA). All constructions were checked by sequencing before cloning them into the binary vector pMDC099 (Curtis and Grossniklaus, 2003) using Gateway™ LR recombination. Constructs were introduced into a Arabidopsis–*eif4e1* ^*KO*^—genome using Floral Dip agrotransformation (Clough and Bent, 1998). Transformants were selected on MS plates supplemented with 10 mg/L hygromycin B.

For the CRISPR‐nCas9‐cytidine deaminase experiments, a 311 nucleotide fragment was synthesized by IDT (Integrated DNA Technologies, Coralville, IA; Table S2) and subcloned into pDONR207 using Gateway™ BP recombination. This fragment inserts a *Bst*XI *Spe*I gene fusion spanning the 3′ end of the AtU6‐26 promoter, a 20 nt long eIF4E1 target and the sgRNA, into the pDICAID_nCas9‐PmCDA_NptII_Della (Shimatani *et al*., 2017). The resulting construct pDICAID_nCas9‐PmCDA_NptII_eIF4E1 was transformed into Col‐0 plants. Transformants were selected on 100 mg/L kanamycin.

### Plant genotyping

A 281 bp sequence encompassing the *eIF4E1* CRISPR target was amplified with specific primers and Sanger sequenced (Table S2).

Segregation of the T‐DNA harbouring the nCas9‐PmCDA_NptII_eIF4E1 construct was carried out by multiplex genotyping of the *nptII* and *eIF4E1* genes as a reference (Table S2). T3 and T4 progenies devoid of this transgene were confirmed as susceptible to kanamycin selection.

High‐resolution melting (HRM) analysis was conducted using the Precision Melt Supermix (Bio‐Rad) according to the manufacturer's recommendations on a 96‐well C1000 Touch™ thermal cycler (Bio‐Rad, Hercules, CA) with two specific primers amplifying a 87 bp fragment spanning the Cas9‐cytidine deaminase target region on *eIF4E1* (Table S2). PCR conditions included an initial denaturation at 95 °C for 2 min, 40 cycles of denaturation at 95 °C for 10 s and annealing/extension at 58 °C for 30 s. This was followed by a melting curve analysis in which heteroduplex sequences formed by raising the temperature to 95 °C for 30 s and lowering it to 60 °C for 60 s. HRM analysis was then carried out by raising the temperature from 65 to 95 °C at 0.2 °C increments. Melting curves were obtained using the Precision Melt Analysis Software (Bio‐Rad).

The Kompetitive Allele Specific PCR (KASP) assay was performed according to the instructions of the KASP genotyping chemistry kit with primers designed to specifically amplify alleles having a C‐to‐G mutation at position +1147 of the *eIF4E1* genomic sequence (LGC, www.lcggroup.com). Thermal cycling was done on an Eppendorf MasterCycler Nexus using the following program: 94 °C for 15 min, 10 cycles of 94 °C for 20 s followed by 65–57 °C for 60 s, decreasing by 0.6 °C per cycle; then 26 cycles of 94 °C for 20 s followed by 55 °C for 60 s. Endpoint detection of fluorescence was performed using an EnVision plate reader (Perkin/Elmer, Waltham, MA).

### Virus materials and resistance assay by DAS‐ELISA

Virus materials used in this study were the following : the ClYVV No. 30 isolate (Sato *et al*., 2005), the ClYVV DSMZ PV0367 isolate (Leibniz Institute DSMZ, Braunschweig, Germany) two distant ClYVV isolates with a 85% homology based on a reference P3 sequence, the WMV Fr isolate (Desbiez and Lecoq, 2004), the TuMV CDN1 isolate (Duprat *et al*., 2002) and the GFP‐fused resistance‐breaking (RB) TuMV plasmid constructions (Bastet *et al*., 2018). Prior to the resistance assay, viruses were propagated on tobacco (*Nicotiana benthamiana*; ClYVV isolates), turnip *Brassica rapa* (TuMV‐CDN1) and zucchini squash *Cucurbita pepo* (WMV‐Fr). Mechanical inoculation using sap was then performed on young leaves of 1‐month‐old Arabidopsis plants (Gallois *et al*., 2010). Viral accumulation of ClYVV isolates, TuMV‐CDN1 and WMV‐Fr was detected on whole rosettes using commercial antibody kits for the DAS‐ELISA assay, directed against ClYVV (Leibniz Institute DSMZ), potyvirus group (Agdia, Elkhart, IN) and WMV (Sediag, Longvic, France) following the manufacturer's protocols. Biological repeats are presented in Figures S6 and S8.

GFP‐fused RB‐TuMV plasmids were multiplied, inoculated and detected following the same procedure described previously (Bastet *et al*., 2018). Briefly, plasmids were multiplied in *Agrobacterium tumefaciens*, plants were agro‐inoculated at 1 month of age by rub‐inoculation on young leaves and viral accumulation was assessed using a closed fluorometric camera FluorCam FC 800‐C/1010‐GFP (Photon System Instruments, Drasov, Czech Republic) equipped with a GFP filter. Fluorescence was represented in false colours.

### Phenotype analyses: bolting time, dry weight, fresh weight and fertility rate

Bolting time analyses started following the transfer of plantlets to soil. Appearance of a 5 mm flowering stem was accounted as the bolting time for 16 plants for each genotype.

Dry and fresh weights were assessed on the same set of 3‐week‐old plants, with at least 26 plants per genotype. For fresh weight, aerial parts were cut and weighed before being dried in a 100 °C heating chamber for 24 h and weighed again to evaluate dry weight.

Fertility rate was assessed based on the total seed production of each plant. The seeds of 10 plants from each genotype were collected and weighed. A set of 100 seeds from each genotype were also weighed to ensure that individual seed mass was similar between genotypes.

Biological repeats are presented in Figures S5 and S7.

### Reverse‐transcription PCR analyses

Total RNA extraction was performed on leaves of 4‐week‐old plants using a TRI‐Reagent solution (Sigma‐Aldrich). For each sample, 1 μg of RNA was used in an RT‐PCR amplification using AMV reverse transcriptase (*Avian myeloblastosis virus*, Promega, Madison, WI) and oligo‐(dT)18 primers. Amplification of cDNAs of *eIF4E1* (At4g18040) and *ADENINE PHOSPHORYBOSYL TRANSFERASE 1* (APT1, At1g27450) for control was done using primers Z3135‐F/Z3135‐R and Z1734/Z1735 respectively (Table S2).

### Western blot analyses

Total protein extracts were prepared from 4‐week‐old plants by grinding leaves in Laemmli buffer and boiling samples for 5 min. The same amount of protein extracts were migrated on electrophoresis gel (SDS‐PAGE) before being transferred to Amersham™ Protran Premium nitrocellulose membranes (GE Healthcare, Chicago, IL). Membranes were then stained with Ponceau S solution (Sigma‐Aldrich) to assess equal loading and correct transfer. Incubation with antibodies directed against actin (1 : 5000 dilution) or eIF4E (1 : 2000 dilution, obtained from Estevan *et al*., 2014) was performed. Secondary antibody incubation was carried out using goat horseradish peroxidase‐labelled anti‐mouse serum for actin and anti‐rabbit serum for eIF4E from Sigma‐Aldrich at the same dilutions as the primary antibodies. Peroxidase activity was then checked using LumiGLO Reserve chemiluminescent substrate kit (KPL, www.kpl.com) and X–OMAT LS films (Kodak, Rochester, NY).

### Cap‐binding assay

Protein extraction was done by grinding young leaves of 4‐week‐old plants and resuspending the powder in a buffer containing 40 mm HEPES/KOH pH 7.6, 100 mm KCl, 1 mm dithiothreitol, 10% glycerol, 1% phenylmethanesulphonylfluoride (PMSF) and a protease inhibitor cocktail (Roche, Basel, Switzerland). After centrifugation at 15,000 ***g*** for 10 min, supernatants (input fraction) were collected and added to 7‐methyl‐GTP sepharose beads (GE Healthcare) following the manufacturer's instructions. After overnight incubation at 4 °C, samples were washed four times with the resuspension buffer described above, followed centrifugation for 1 min at 15,000 ***g*** at 4 °C. Pellets were collected and elution of proteins bound to cap analogue beads (output fraction) was done by preparing samples following the procedure described for western blot analyses. Western blots were thereafter performed to detect eIF4E1 and actin proteins on output and input (total proteins) fractions respectively.

### Statistical analyses

All data in this publication were tested using a Kruskal–Wallis statistical test to determine significant differences compared to the wild type, using the pgirmess package in the free software R (https://www.r-project.org/).

## Conflict of interest

The authors declare no conflict of interest.

## Supporting information


**Figure S1** Surface hydrophobicity potential of the eIF4E1 proteins encoded by the five constructed alleles *eIF4E1* ^*W69L*^, *eIF4E1* ^*T80DS81D*^, *eIF4E1* ^*S84A*^, *eIF4E1* ^*G114R*^ and *eIF4E1* ^*N176K*^ compared to the wild‐type eIF4E1.

**Figure S2** Analysis of the correct eIF4E1 transgenes expression in transformed plants.

**Figure S3** Phenotype analysis of double‐mutant *eif4e1* ^*KO*^ *eifiso4e* ^*KO*^ plants complemented with *eIF4E1* ^*T80DS81D*^, *eIF4E1* ^*G114R*^ or *eIF4E1* ^*N176K*^ alleles.

**Figure S4** Analysis of *eIF4E1* ^*N176K*^ expression in T4 CRISPR‐Cas9 cytidine deaminase modified transgene‐free plants.

**Figure S5** Biological repeat. Functional *in planta* complementation of the *eif4e1* knock‐out by the five constructed alleles *eIF4E1* ^*W69L*^, *eIF4E1* ^*T80DS81D*^, *eIF4E1* ^*S84A*^, *eIF4E1* ^*G114R*^ and *eIF4E1* ^*N176K*^.

**Figure S6** Biological repeat. Viral accumulation of ClYVV in *eif4e1* ^*KO*^ plants complemented with constructed alleles assessed using DAS‐ELISA.

**Figure S7** Biological repeat. Viability and phenotype assessment of double‐mutant *eif4e1* ^*KO*^ *eifiso4e* ^*KO*^ plants complemented with *eIF4E1* ^*T80DS81D*^, *eIF4E1* ^*G114R*^ or *eIF4E1* ^*N176K*^ alleles.

**Figure S8** Biological repeat. Virus resistance analyses of *eIF4E1* ^*T80DS81D*^, *eIF4E1* ^*G114R*^ or *eIF4E1* ^*N176K*^ alleles in a double‐mutant *eif4e1* ^*KO*^ *eifiso4e* ^*KO*^ background.

**Table S1** Base changes resulting from the genome editing of the cytosine 1447 of eIF4E1 in T2 and T3 plants obtained from T1 plants harbouring the pDICAID_nCas9‐PmCDA_NptII_eIF4E1 construct.

**Table S2** List of oligonucleotides used in this study.
