# Supplementary figures and images for: Mimicking natural polymorphism in eIF4E by CRISPR‐Cas9 base editing is associated with resistance to potyviruses
Source: Plant Biotechnol J. 2019 Mar 5;17(9):1736–50. doi: 10.1111/pbi.13096 (PMC6686125; doi:10.1111/pbi.13096)

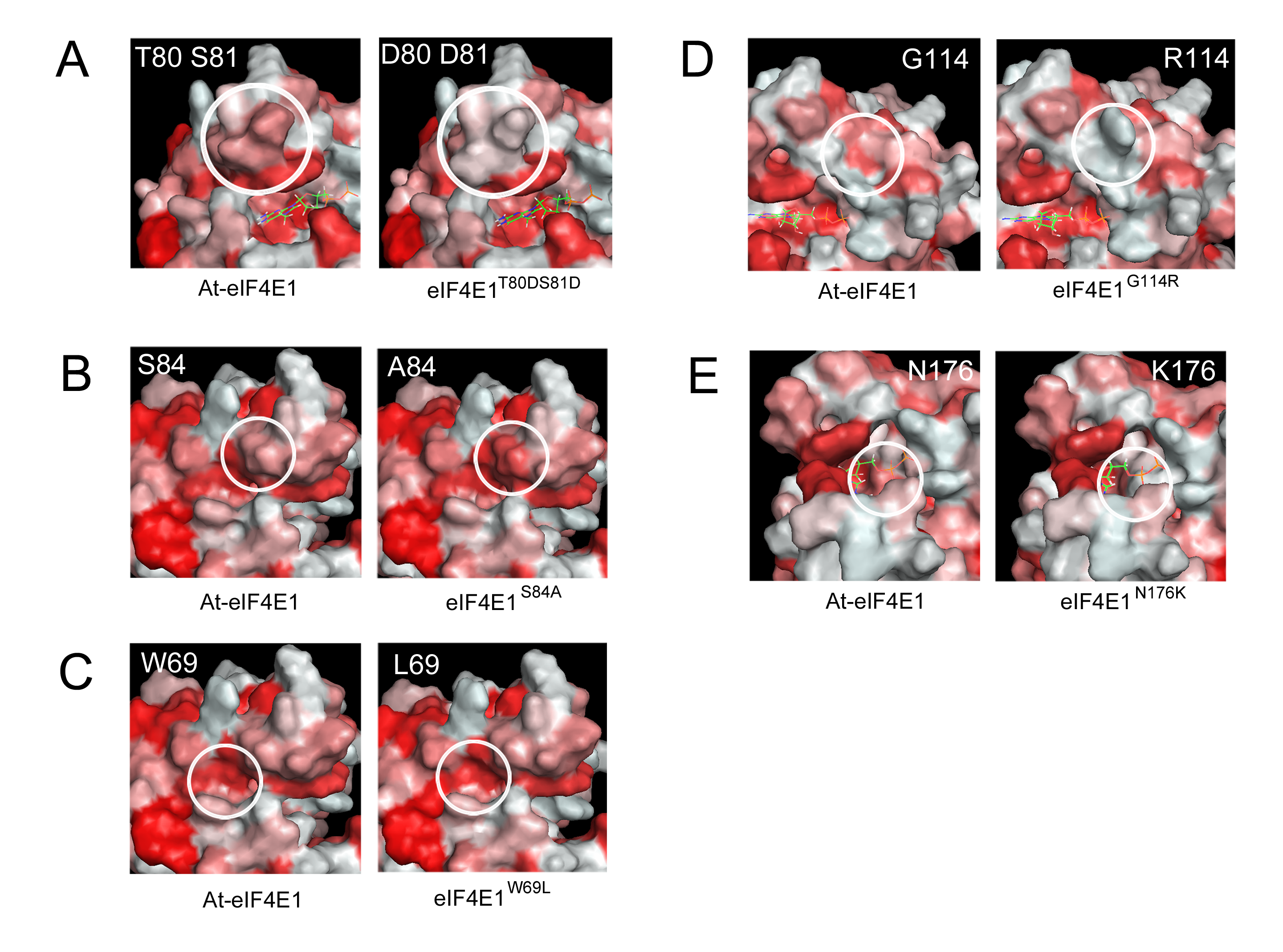

Supplement: Supplementary file 1 — Figure S1 Surface hydrophobicity potential of the eIF4E1 proteins encoded by the five constructed alleles eIF4E1 W69L , eIF4E1 T80DS81D , eIF4E1 S84A , eIF4E1 G114R and eIF4E1 N176K compared to the wild‐type eIF4E1. [file PBI-17-1736-s005.tif]

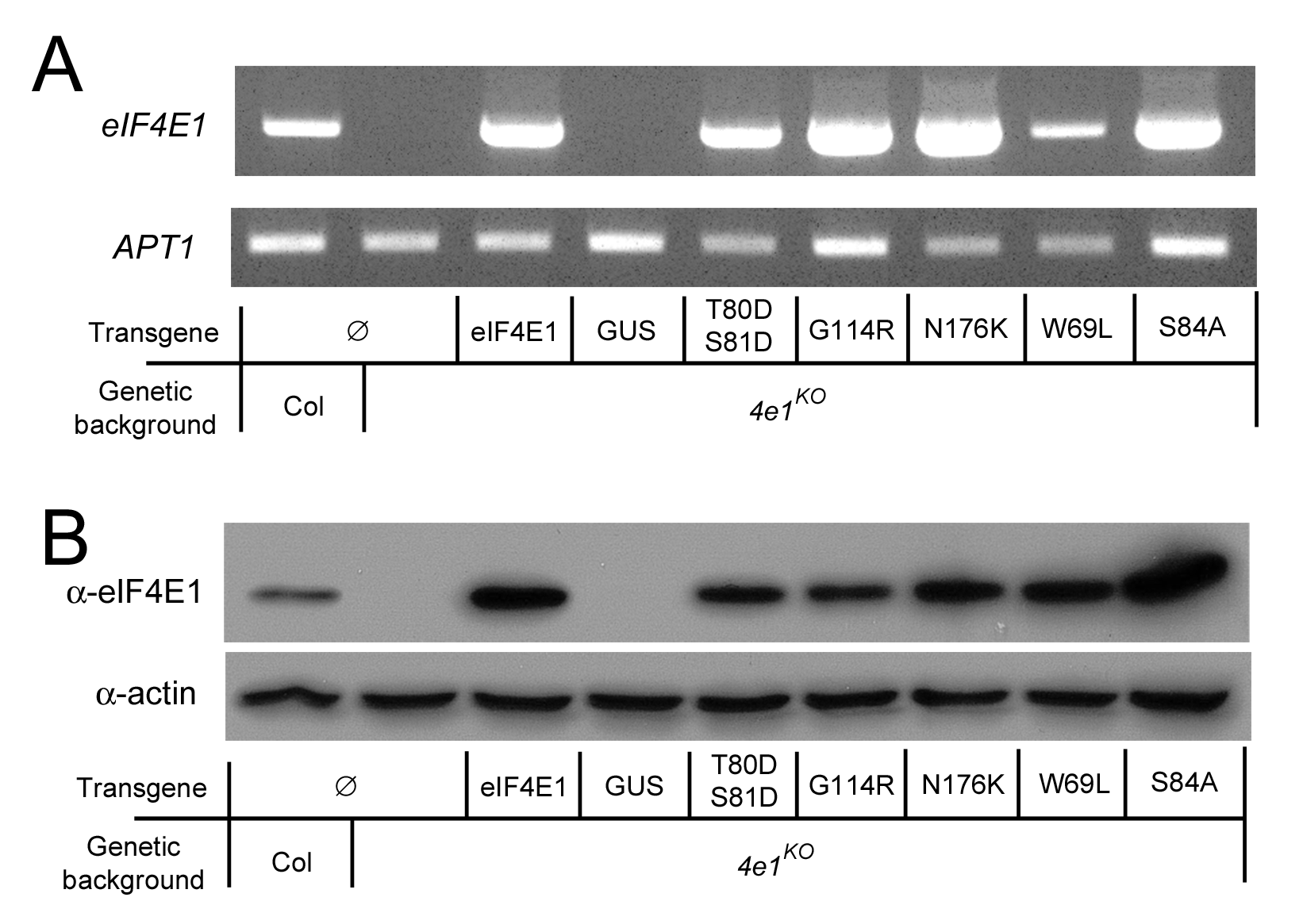

Supplement: Supplementary file 2 — Figure S2 Analysis of the correct eIF4E1 transgenes expression in transformed plants. [file PBI-17-1736-s008.tif]

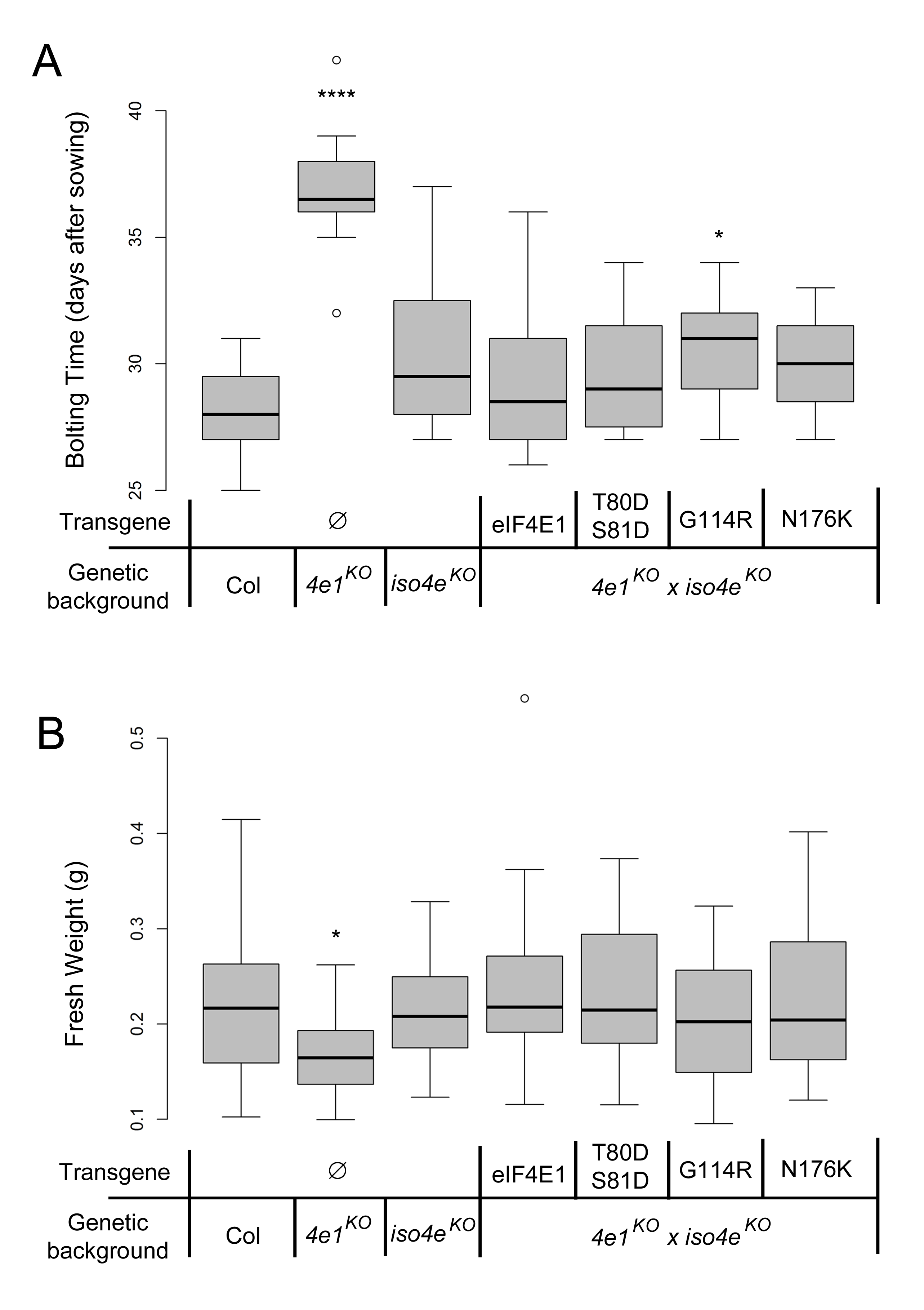

Supplement: Supplementary file 3 — Figure S3 Phenotype analysis of double‐mutant eif4e1 KO eifiso4e KO plants complemented with eIF4E1 T80DS81D , eIF4E1 G114R or eIF4E1 N176K alleles. [file PBI-17-1736-s007.tif]

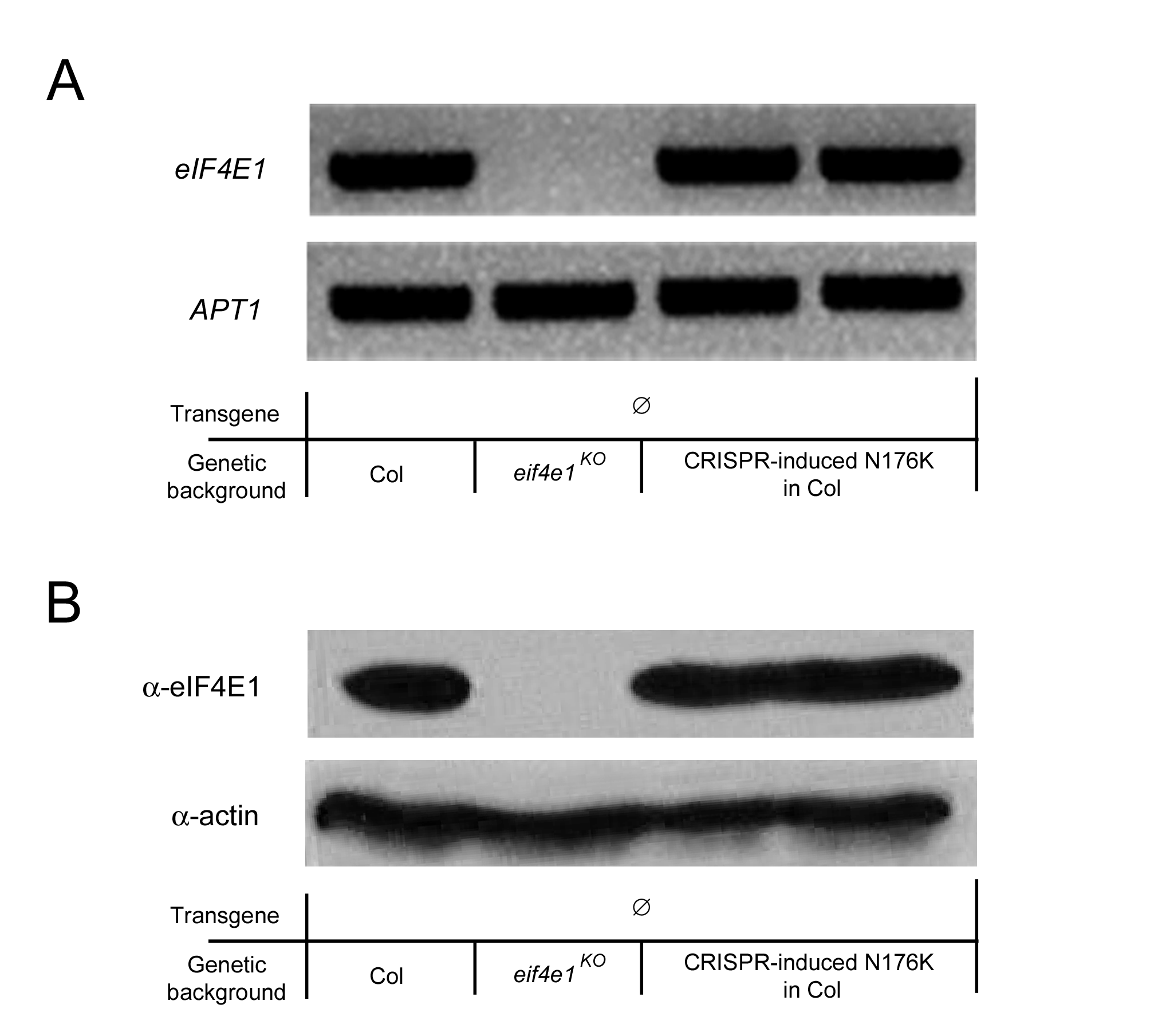

Supplement: Supplementary file 4 — Figure S4 Analysis of eIF4E1 N176K expression in T4 CRISPR‐Cas9 cytidine deaminase modified transgene‐free plants. [file PBI-17-1736-s009.tif]

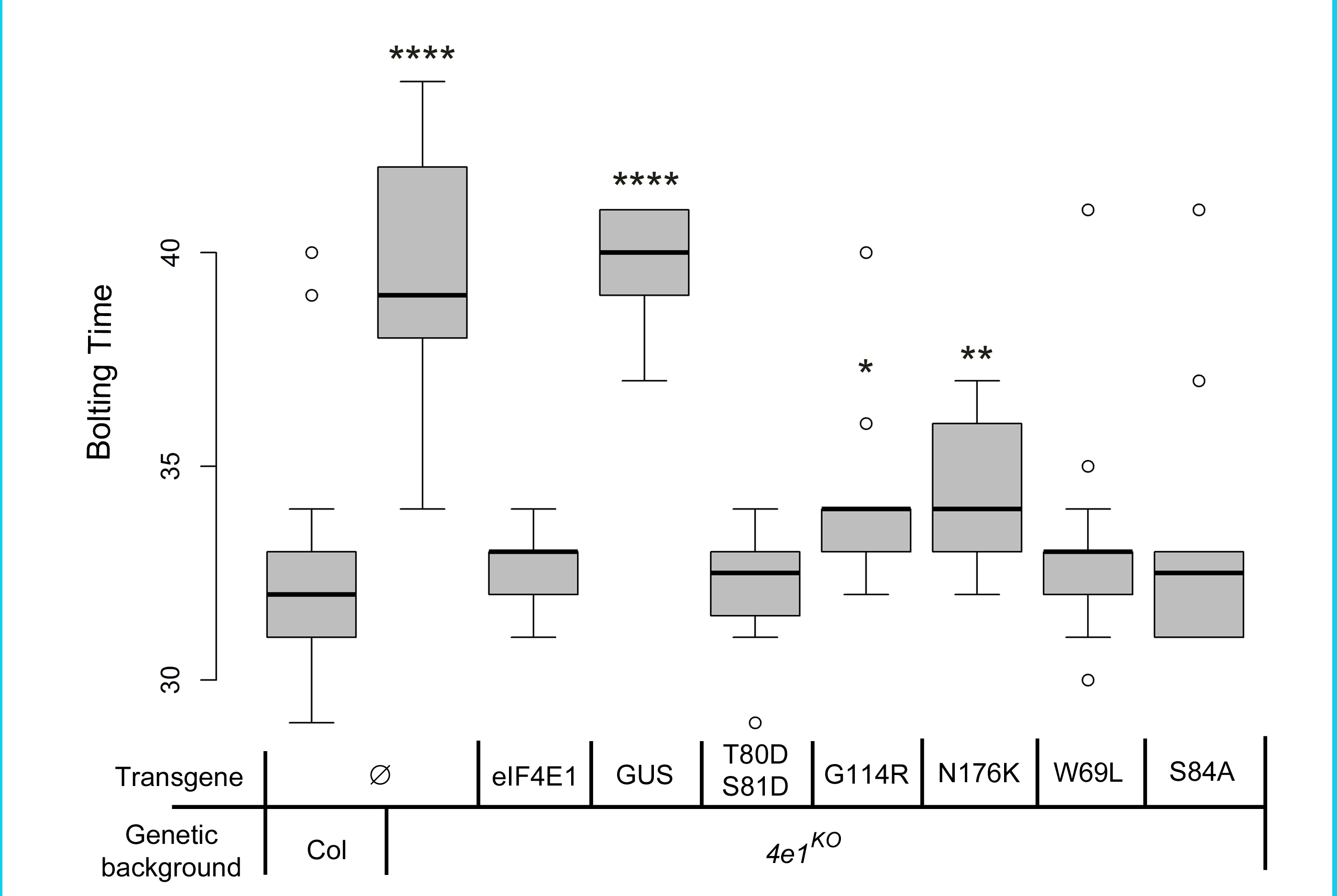

Supplement: Supplementary file 5 — Figure S5 Biological repeat. Functional in planta complementation of the eif4e1 knock‐out by the five constructed alleles eIF4E1 W69L , eIF4E1 T80DS81D , eIF4E1 S84A , eIF4E1 G114R and eIF4E1 N176K . [file PBI-17-1736-s001.tif]

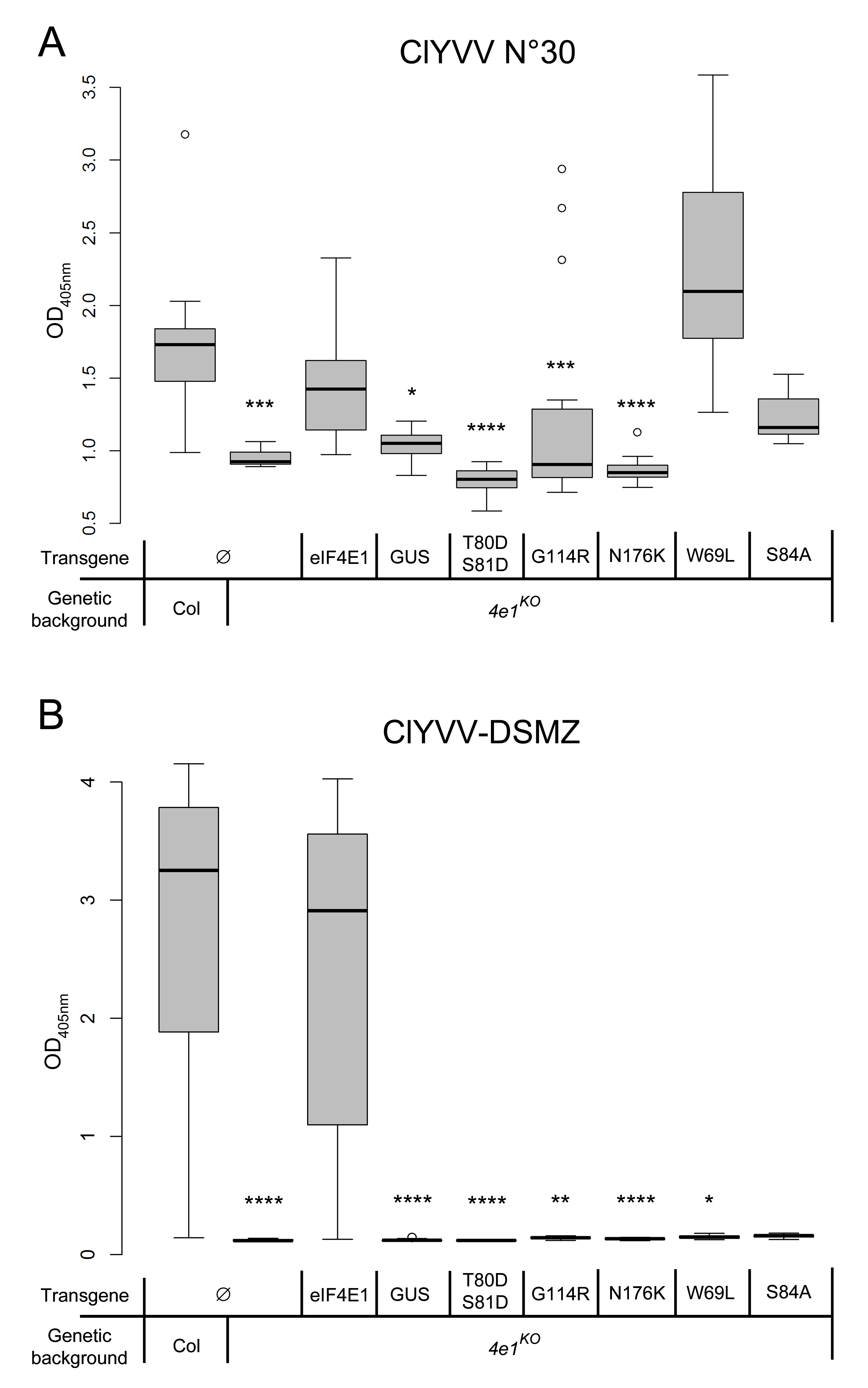

Supplement: Supplementary file 6 — Figure S6 Biological repeat. Viral accumulation of ClYVV in eif4e1 KO plants complemented with constructed alleles assessed using DAS‐ELISA. [file PBI-17-1736-s002.tif]

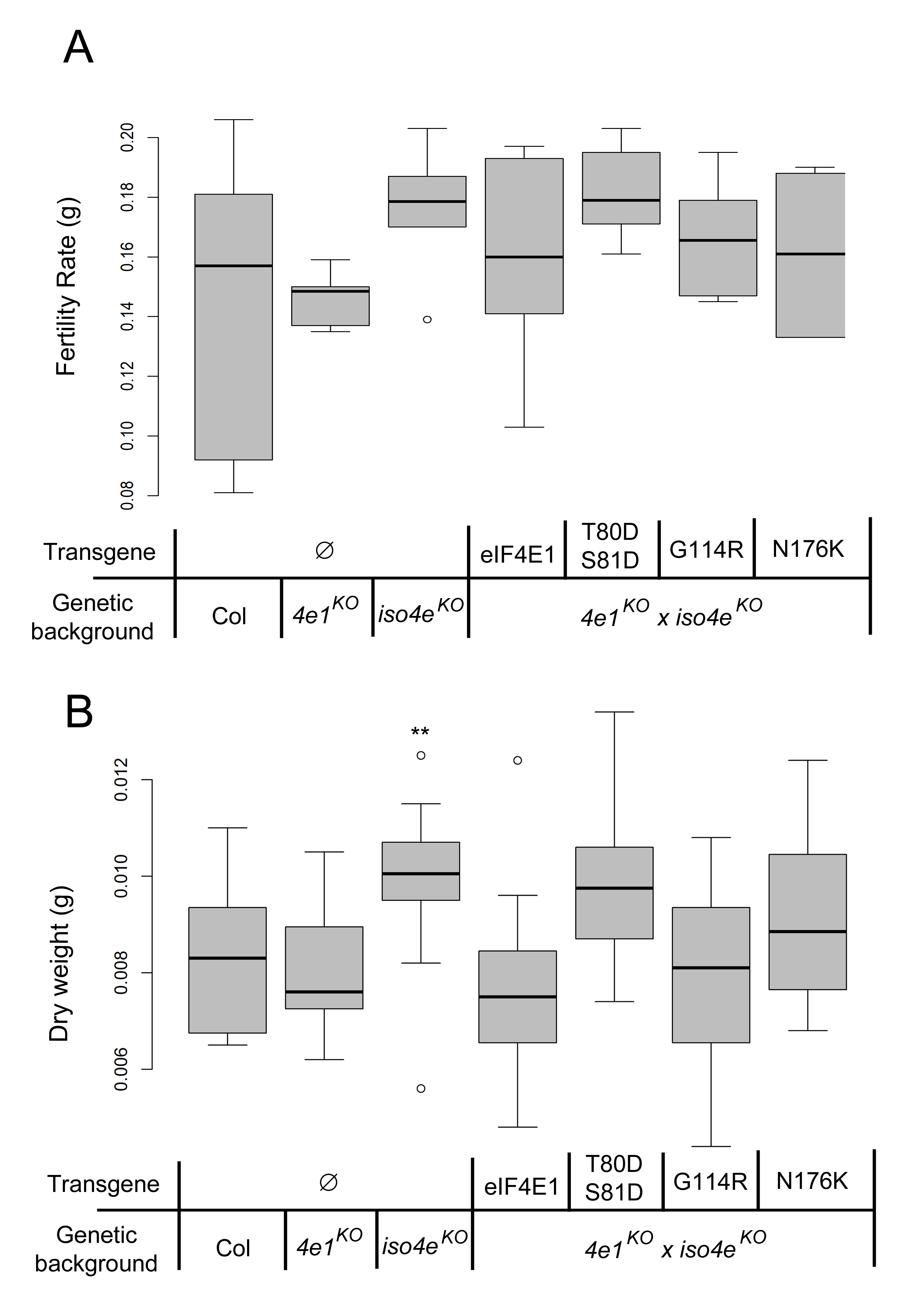

Supplement: Supplementary file 7 — Figure S7 Biological repeat. Viability and phenotype assessment of double‐mutant eif4e1 KO eifiso4e KO plants complemented with eIF4E1 T80DS81D , eIF4E1 G114R or eIF4E1 N176K alleles. [file PBI-17-1736-s003.tif]

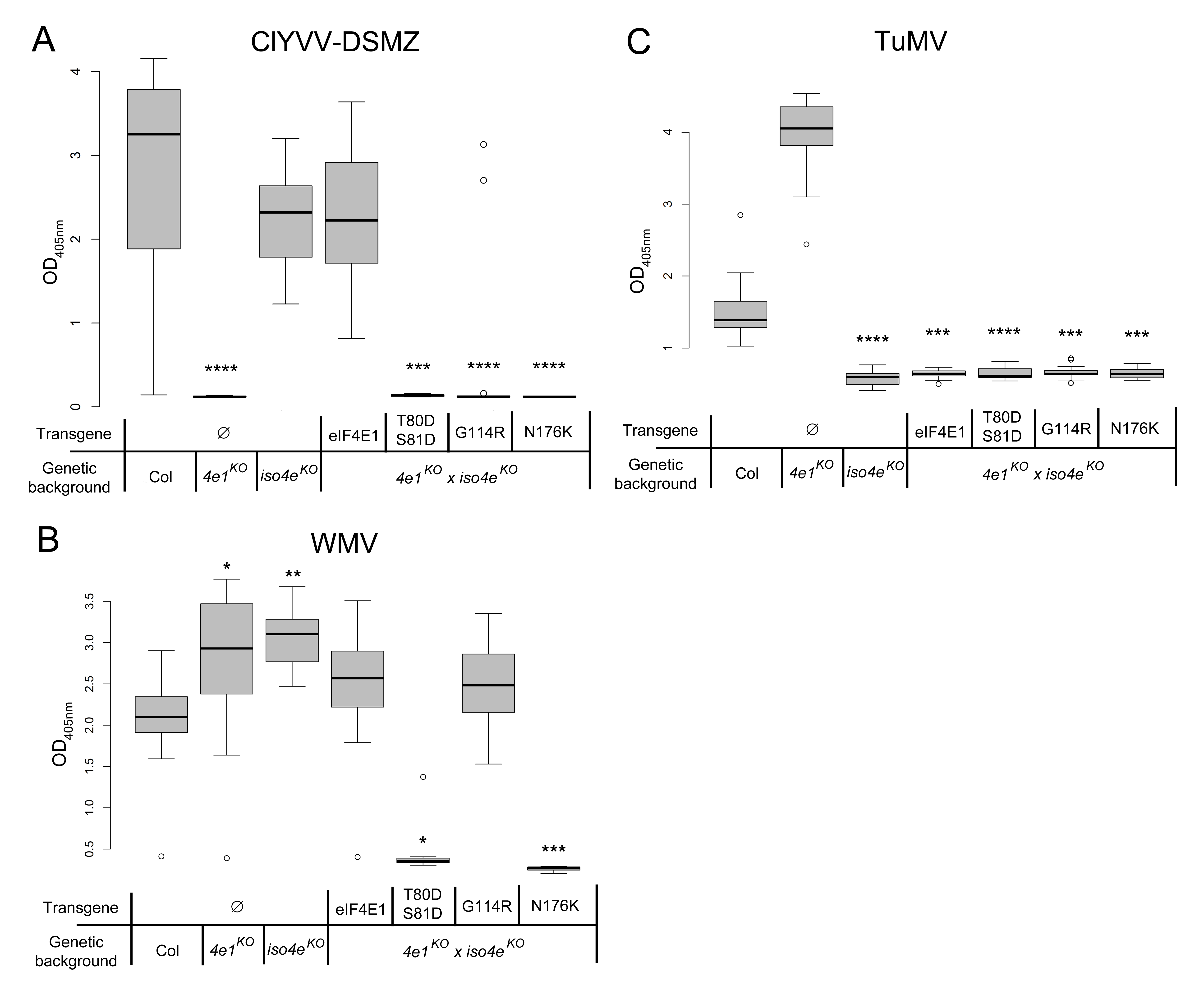

Supplement: Supplementary file 8 — Figure S8 Biological repeat. Virus resistance analyses of eIF4E1 T80DS81D , eIF4E1 G114R or eIF4E1 N176K alleles in a double‐mutant eif4e1 KO eifiso4e KO background. [file PBI-17-1736-s004.tif]
