# Supplementary material for: Mimicking natural polymorphism in eIF4E by CRISPR‐Cas9 base editing is associated with resistance to potyviruses
Source: Plant Biotechnol J. 2019 Mar 5;17(9):1736–50. doi: 10.1111/pbi.13096 (PMC6686125; doi:10.1111/pbi.13096)
Supplement: Supplementary file 9 — Table S1 Base changes resulting from the genome editing of the cytosine 1447 of eIF4E1 in T2 and T3 plants obtained from T1 plants harbouring the pDICAID_nCas9‐PmCDA_NptII_eIF4E1 construct. [file PBI-17-1736-s010.pdf]

Base at + 1447 position	T2 plants	T3 plants
C (unchanged)	2	4
G	1	-
C, G	1	1
C, T	-	1
G, T	-	2
> 2 bases	9	8
Indels	2	-

Table S1. Base changes resulting from the genome editing of the Cytosine 1447 of *eIF4E1* in T2 and T3 plants obtained from T1 plants harboring the pDICAID_nCas9-PmCDA_NptII_eIF4E1 construct. Base status was characterized locus based on the chromatograms following Sanger sequencing of the *eIF4E1*.
